# Open source data logger for low-cost environmental monitoring

**DOI:** 10.3897/BDJ.2.e1059

**Published:** 2014-02-11

**Authors:** Ed Baker

**Affiliations:** † The Natural History Museum, London, United Kingdom

**Keywords:** Arduino, open source, open hardware, environmental monitoring, big data, internet of things, temperature, humidity, Ethernet

## Abstract

The increasing transformation of biodiversity into a data-intensive science has seen numerous independent systems linked and aggregated into the current landscape of biodiversity informatics. This paper outlines how we can move forward with this programme, incorporating real time environmental monitoring into our methodology using low-power and low-cost computing platforms.

## Introduction

Low power and cheap computational projects such as Arduino and Raspberry Pi have brought the use of small computers and micro-controllers to the masses, and their use in fields related to biodiversity science is increasing (e.g. this presentation by Hirafuji shows the use of Arduino in agriculture). There is a large amount of potential in using automated tools for monitoring environments and identifying species based on these emerging hardware platforms, but to be truly useful we must integrate the data they generate with our existing systems. This paper describes the construction of an open source environmental data logger based on the Arduino platform and its integration with the web content management system Drupal which is used as the basis for Scratchpads (Smith et al. 2011) among other biodiversity tools. The Drupal platform uses familiar open source standards while the Arduino hardware uses the emerging open hardware licences (see below). It is assumed that most biodiversity scientists are not familiar with the construction of electronic devices, so this paper is presented more in the style of a 'how to guide' rather than a straightforward functional description. It is hoped that people will make use of the technologies described here and adapt them to the needs of their own interests and projects.

### Open hardware?

The open source hardware movement extends the familiar licences of open access and open source software to physical objects. The aim is to create an ecosystem of projects and products much like the community that develops around open source software. To be open hardware, all materials needed to construct the hardware (schematics, printed circuit board (PCB) layouts, bills of materials) and the software required (on device and ideally to interface with the device) must be released under a permissive 'copyleft' licence. More information about open hardware can be found at the Open Source Hardware Association.

### Citizen Engineers

The introduction of easy-to-use micro-controller devices such as the Arduino brought working with digital electronics to a broader audience. The Arduino project (and similar initiatives) has generated a number of books and a plethora of web pages detailing the basic use of the devices and the use of sensors (ideal for data-logging), writing to SD cards (Secure Digital: a format of memory card widely used in digital cameras) and interfacing with the Internet (e.g. McRoberts (2010), McEwen and Cassimally (2014). Additionally, there are a wide range of books describing the basics of electronics, Scherz (2007) is recommended as a reference text due to its comprehensive approach and detailed theory but one of the many electronics 'primer' books may be a better introduction. There is great potential for the biodiversity community if we collaborate with knowledgeable 'hackers' and 'makers' as citizen engineers in the same way we interact with knowledgeable amateur natural historians as citizen scientists.

## Project description

### Design description

The device constructed is intended both as a functional device capable of generating useful environmental datasets and also as a demonstration to the biodiversity community of what is possible using the Arduino system as a base. This project aims to be as easy as possible to construct and use for those with no experience in electronic devices, and for this reason we have steered away from the use of custom PCBs or other materials. The use of a prototyping board rather than a custom PCB also allows for the project to be extended more easily and assembled without soldering. The author would encourage anybody who wishes to release a custom PCB layout for this project to do so.

### Funding

This project was supported by the European Union funded ViBRANT project (Contract no. RI-261532).

## Web location (URIs)

Homepage: http://ebaker.me.uk/project-role/open-source-data-logger

Bug database: https://github.com/edwbaker/environment-data-logger/issues

## Technical specification

Platform: Arduino / Drupal

Programming language: C / PHP

Operational system: Arduino / Linux (can also run on Windows/OS X)

## Repository

Type: Git

Browse URI: https://github.com/edwbaker/environment-data-logger

## Usage rights

### Use license

Other

### IP rights notes

The Drupal code and Arduino code are released under the GNU General Public License version 2 or later.

## Implementation

### Implements specification

The requirements for the device, and the server software (Drupal) that receives the data were specified as follows:

**Data Logger**

- Be able to record the temperature and humidity at a regular time interval (e.g. every 5 minutes)
- Record the data collected to a micro-SD card
- Submit data collected automatically and in real time to a Drupal website (using an Ethernet connection)
- Have a real time clock (RTC) – the Arduino internal clock counts milliseconds since the micro-controller was turned on, we need to record the actual time of measurement
- Use standard and readily-available components
- Be able to run off readily-available batteries (e.g. AA) for at least 8 hours

**Server software**

- Be able to receive data from a device or more than one device and save it as a Drupal node (content item)
- Be able to generate code for the Arduino to assist others in using this software to post data

### Audience

This project should be useful to a wide range of biodiversity scientists, some possible study scenarios are

- using a single device alongside a light, malaise or pitfall trap to collect abiotic data over the study period
- using multiple devices to study small climatic differences between different micro-habitats
- using devices over a long period to compliment studies on organism phenology

It is hoped that the publication of this device will encourage biodiversity scientists to collaborate outside of their discipline, whether it be with citizen engineers or professional academics. A work by Baker, Bennett and Chesmore (University of York) in 2013 (in prep) expands the concept of using electronic devices from monitoring environments to automated species identification using the acoustics of orthoptera. Where reliable identification of species can be performed by machines, the potential for collecting high quality, high precision and long-term datasets on species abundance alongside environmental variables becomes a possibility.

## Additional information

### Sourcing of components

This project uses standard components which can be sourced from specialist electronics suppliers (e.g. Maplin, RS, Rapid Electronics in the UK). A number of online shops serve the needs of the hacker and maker communities including Adafruit in the US and Cool Compnents in the UK. Generic online retailers such as eBay and Amazon will also have listings for most, if not all, of the materials required.

### Assembly of the device

**Figure 1.**
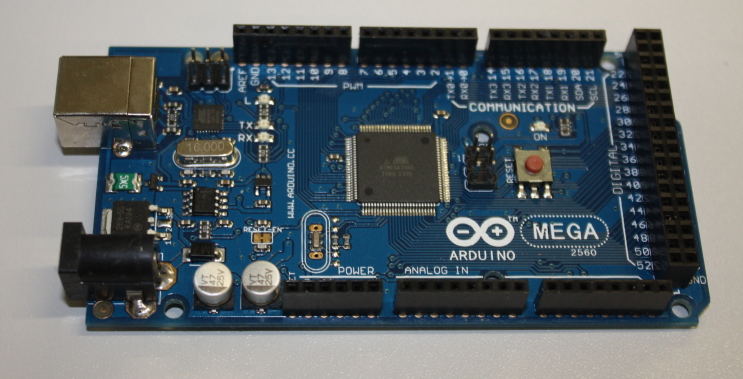
An Arduino Mega board. The board can be powered and programmed using the USB connector (top left) or powered as a standalone device using the circular power connector (bottom left).

The data logging device uses the Arduino Mega board (Fig. 1) which encompasses an ATmega2560 micro-controller and associated hardware to provide a clock signal, regulate power to the board and to allow the micro-controller to be programmed via the USB interface. A number of analogue input and digital input/output pins are exposed on the board for the attachment of other electronic devices for input or output (these connectors are known as 'headers'). The Arduino Mega is an expanded version of the standard Arduino Uno which has a greater number of input/output connections and also a larger programmable memory. The code and libraries required for this project exceed the Arduino Uno's capacity, but an Arduino Uno may be used if some functionality (e.g. the real-time clock or Ethernet libraries) is removed. You will need to download the Arduino Integrated Development Environment (IDE) software from the Arduino website. The Arduino platform is favoured over the Raspberry Pi in this instance due to its lower power consumption allowing the device to run longer on battery power (the Raspberry Pi is a low-power Linux computer and has capabilities well beyond what is needed for this project).

**Figure 2.**
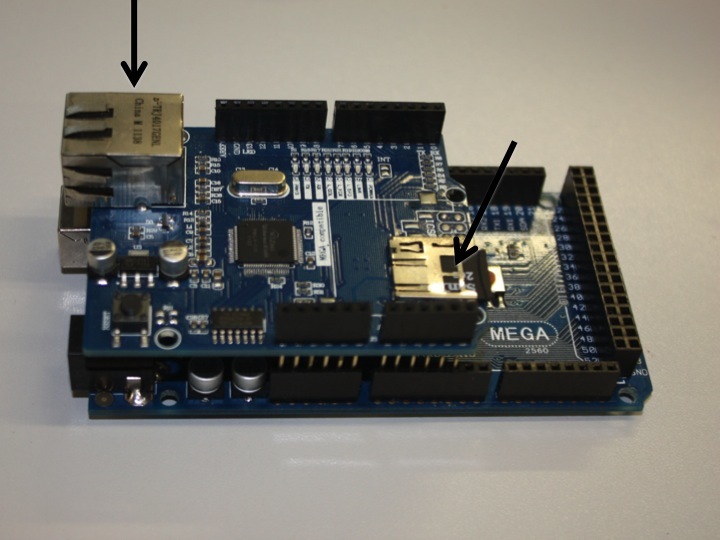
The Arduino Ethernet Shield attached to the Arduino Uno. The Ethernet socket (left) and micro-SD socket (right) are indicated.

**Figure 3.**
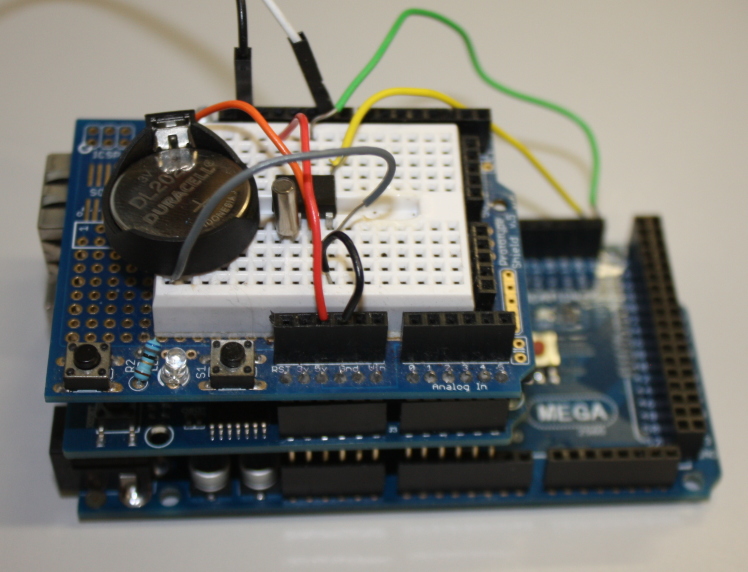
The Arduino Mega (bottom), Ethernet shield (middle) and prototyping shield (top) stacked as the core of the data logger. Some of the other components of the device are being assembled using the solder-less breadboard on the prototyping shield.

Several expansion boards are available for the Arduino, which have pins allowing them to attach to the black connectors on the main Arduino board. These expansion boards are known as 'shields' and two different ones have been used in this project. The first, the Arduino Ethernet Shield (Fig. 2), provides both an Ethernet interface (allowing the Arduino to communicate over a local network or the Internet) and allows a micro-SD card to be inserted (allowing data to be saved locally). Both of these shields expose the full set of Arduino headers – allowing the shields to be stacked on each other for additional functionality. Standard software libraries for the Arduino are available to provide easy access in code to this hardware. The second shield used is one of the available 'prototyping shields' – an electronics breadboard that allows for the prototyping of different circuitry without having to make permanent soldered connections (Fig. 3). While for a production product a custom printed circuit board (PCB) would be much better than a temporary prototype shield it is used here, as one of the hopes of this paper is that readers will take the work presented and once familiar with the principles modify or extend it to meet the requirements of their projects.

By stacking first the Ethernet shield and then the prototyping shield on top of the Arduino Mega the central core of the data logger is formed. This central system controls reading data from the sensors, processing that data into standard units of measurement if needed, then recording them to a micro-SD card and/or submitting them to a website. What we have is a small, low power and low cost Internet-connected computational device. Systems using these components are often used for 'Internet of Things' projects (McEwen and Cassimally 2014).

**Figure 4.**
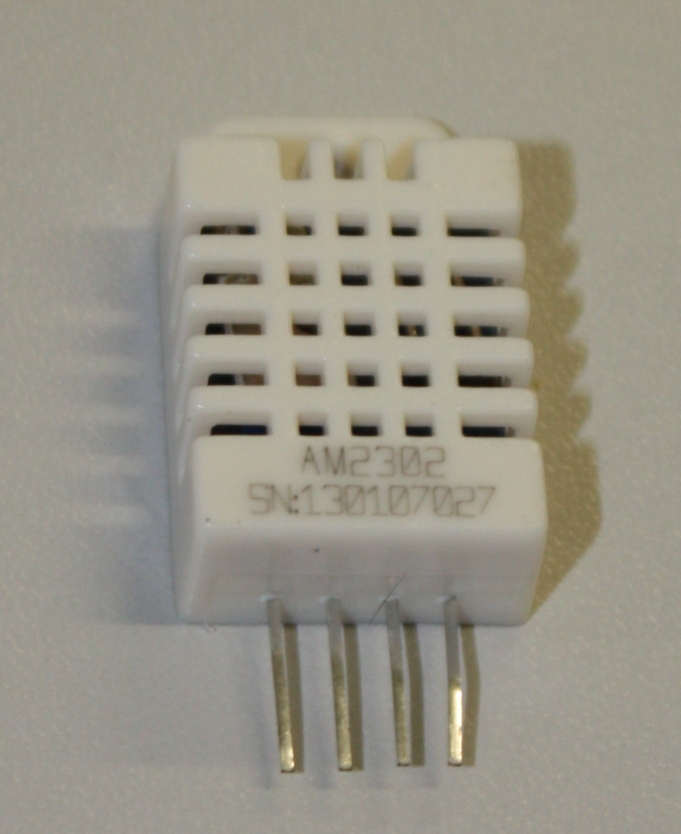
DHT22 digital temperature and humidity sensor in AM2303 package. From left to right the pins are VDD (power supply), DATA, unused and GRD (ground).

**Figure 5.**
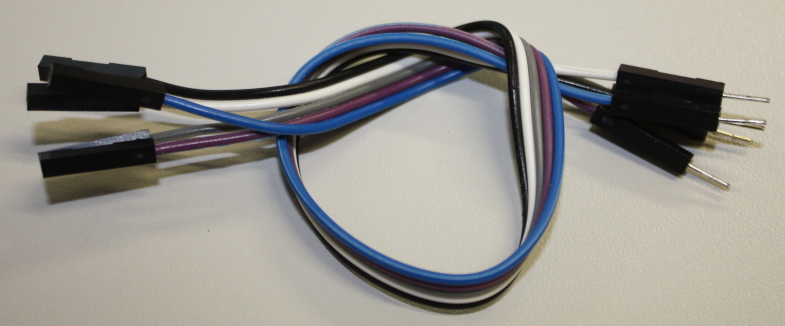
Male-Female connecting wires make it easy to attach sensors to the Arduino board while prototyping.

The temperature and humidity measurements are made using a DHT22 digital sensor (a digital output sensor sold packaged in plastic housing as AM2303; Fig. 4). These sensors communicate with the Arduino using a DHT22 library (this abstracts the details of communicating directly with the sensor). You will need to download this library and add it to your Arduino Integrated Development Environment (IDE) before trying to compile the code for this project (see Installing Additional Arduino Libraries). For ease of (re/de)assembly male-female connecting wires (Fig. 5) are used to attach the sensor to the Arduino. With the sensor facing you the pins are numbered left to right from 1 to 4. Pin 1 should be connected to the 5 V supply from the Arduino, pin 2 from the sensor should be attached to digital (not analogue) pin 2 on the Arduino – this is the line of communication between the devices. Finally pin 4 should be attached to the Arduino ground (GND) – pin 3 is not used.

**Figure 6.**
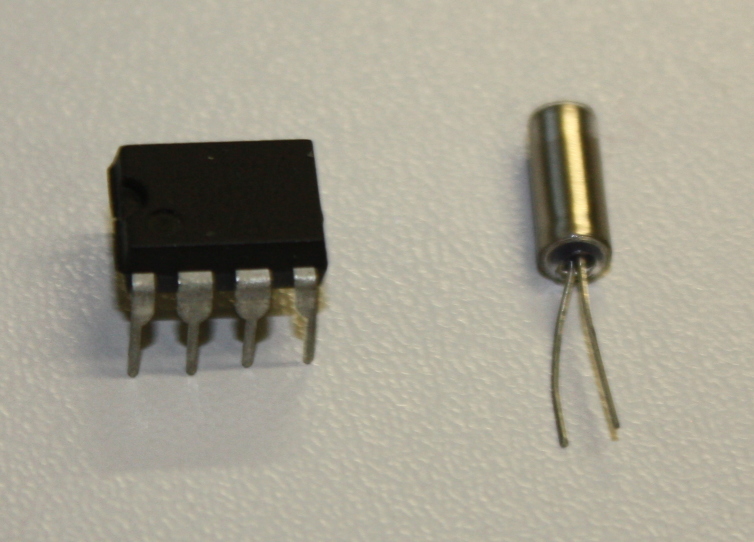
DS1307 RTC and 32.768 kHz crystal.

**Figure 7.**
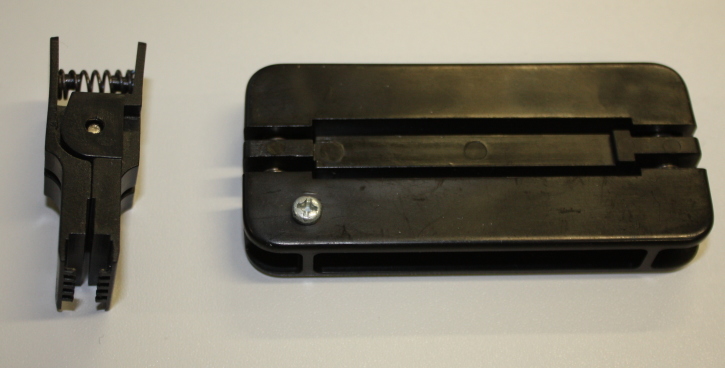
Tools for manipulating DIL integrated circuit packages such as the DS1307. On the left is an extraction tool which helps to remove DIL packaged chips from a breadboard, on the right is a tool to assist in straightening any bent pins on the package.

**Figure 8.**
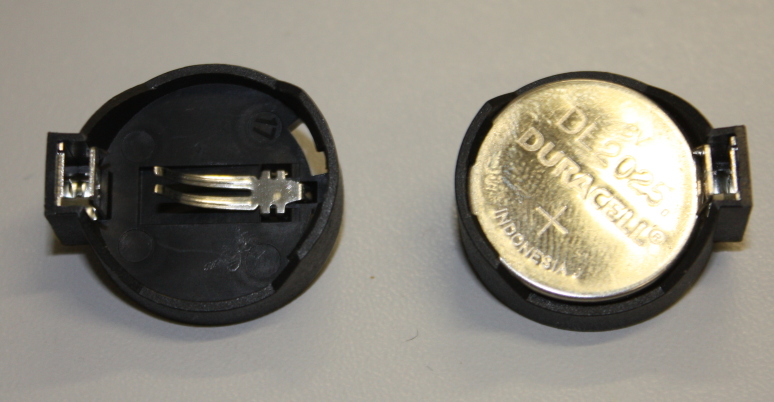
Clip for a Lithium coin cell: empty (left) and with cell inserted (right). The clip has pins for insertion into a breadboard or PCB on the reverse.

The device uses a battery powered real time clock (RTC) to maintain an internal measurement of Universal Coordinated Time (UTC). The RTC chip used is a standard DS1307 in a dual-in line (DIL) package (Fig. 6) and we make use of the DS1307RTC Arduino library. You may prefer to invest in some basic tools for handling DIL packages (Fig. 7). In addition to the DS1307 chip we also need a 32.768 kHz crystal (used by the DS1307 to maintain accurate time; Fig. 6) and a 3 V Lithium cell and cell holder (a CR2032 cell is used here; Fig. 8). These Lithium cells are widely known as "coin" or "watch" cells. The cell provides a power source for the DS1307 and crystal circuit when the Arduino power is removed (when the device is turned off). The DS1307 has very low power requirements and a CR2032 cell should last for several years.

The Arduino uses the I^2^C (Inter-Integrated Circuit) bus to communicate with the DS1307. There is a standard pin numbering system for DIL packages: with the chip pins down and the notch to the left, the bottom left pin is number 1. Pins are numbered anti-clockwise from pin 1, so that on the DS1307 the pin at top left is numbered 8. The 32.768 kHz crystal is connected between pins 1 and 2. The positive connection to the Lithium cell is made to pin 3 (the negative is connected to the Arduino's ground (GND) connector). Pin 4 is connected to the Arduino ground (GND). Pin 5 is connected to the Arduino's SDA connector (pin 20 on the mega) and pin 6 to the Arduino's SCL (pin 21 on the Mega). It should be noted that these connectors are on the Arduino board and are not part of the headers made available via the Ethernet and prototyping shields. SCL stands for 'serial clock' and is used to control when data is sent, the data is sent over the SDA line ('serial data'). Pin 7 of the DS1307 provides a square-wave output that is not used here. Pin 8 should be attached to the Arduino's 5 V. A schematic and photograph of a real circuit are shown in Fig. 9.

In order to set the clock we use the SetTime example sketch that comes with the library (a sketch in Arduino is the source code for the software uploaded to and run on the device). When this sketch is uploaded to the Arduino, it uses the time on the computer programming the Arduino to set the internal clock of the DS1307. Any sketch uploaded after this will be able to request the current time from the DS1307 as long as the Lithium cell is providing backup power. The sketch can be found in the Arduino IDE (once the library has been installed, see above) from File > Examples > DS1307RTC > SetTime. Connect the Arduino to the computer using the USB cable and upload the sketch (program) to the device using the Upload button. The Upload button will compile the sketch and then upload it to the Arduino. As soon as the Arduino has been programmed it will start to run the sketch. As this sketch has no obvious outputs you may wish to open the serial monitor (Tools > Serial Monitor) to view the output. Assuming the hardware has been connected properly (the serial monitor will inform you otherwise) you may then open and upload the other example sketch, ReadTime. ReadTime will output the current date and time, as known to the DS1307, to the serial monitor.

The final non-standard library used by this project is Sleep_n0m1. This library handles putting the Arduino micro-controller into a low power state for a specified number of seconds between readings. This low-power mode will allow the data logger to function for a longer time period between battery changes.

Finally an LED (light emitting dioide) is attached between pin 9 and ground. This will be used to indicate when a reading is made (single flash) or an error condition (rapid blinking).

Now that the hardware assembly is complete it is time to upload the code from this project's GitHub repository to the data logger using the Arduino IDE. Both the edl.ino and lta_struct.h files must be present for the code to compile and function.

The device uses custom error handling routines using a custom C struct called 'error' defined in lta_struct.h (from this project's GitHub repository). A struct in the C programming language is a collection of variables grouped together under a single name (and accessed via a single pointer). When an error is encountered an instance of error is created including a warning message and an integer representing the severity of the error. The function calling the error then parses the error to the function error_condition(error). This function is responsible for the handling of all errors and has the responsibility of deciding how they should be output or recorded (e.g. if we are logging data to an SD card we can append them to an error log file). The most basic output is flashing the LED indicator in a way that indicates an error has occurred (rapid flashes) and the severity of that condition (the number of flashes in quick succession).

The data logger is now fully functional but would not last long in an outdoor setting. In order to make it more weatherproof we must package the components into a suitable enclosure. There are a number of suitable ABS (Acrylonitrile butadiene styrene) plastic enclosures available, the dimensions of the one used here are 165×125×75 mm. When packaging an electronic device there are considerations to be made about how watertight the case can be. Components such as the humidity sensor must be placed outside of the device, and having some components such as a power switch and LED indicator on the outside will prevent having to unscrew the enclosure too regularly. Each device perforating the enclosure is a possible source of weather failure however, so it will likely pay to keep the number of external controls minimal. Risk can be minimised by choosing weatherproof connectors, and where possible sealing other connections using glue or sealant.

**Figure 10.**
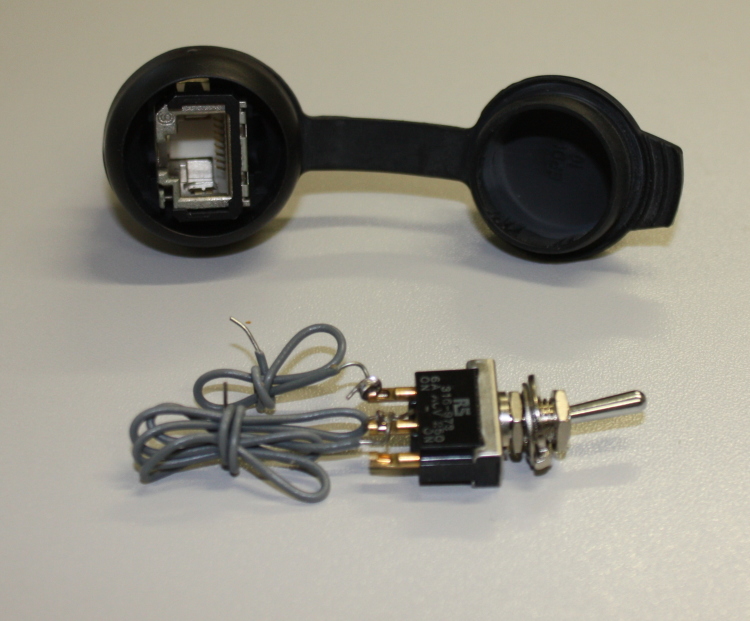
Weatherproof Ethernet panel mount connector (top) and panel mount toggle switch (bottom).

**Figure 11.**
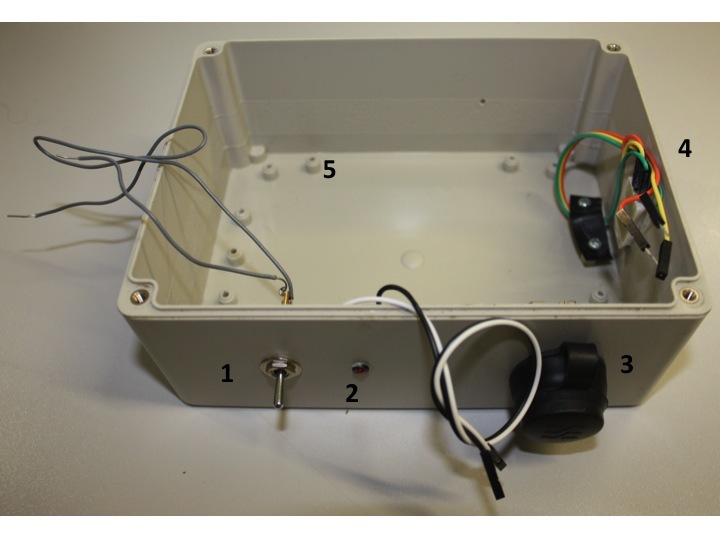
The enclosure with external components mounted.
Single pole single throw toggle switch (panel mount).LED in LED panel mount.Weatherproof panel mount Ethernet connector.Temperature and humidity sensor mounted through enclosure. The cables are routed and secured using film plastic attached to the enclosure with screws.Sockets for screwing components inside the device.

The enclosure will now need to be prepared to receive the device. The Arduino board and battery holder will need to be secured to the enclosure using screws (and the screw mounts in the case) or using self-attaching plastic PCB mounts. Holes will then need to be drilled for the power switch, LED indicator, Ethernet socket and the sensor. The sensor is not circular unlike the other components so will need a hole drilled, and then either expanded using a hacksaw or file to the desired size. The power switch and Ethernet connector (Fig. 10) are both secured using the nut and thread provided. The power switch should be mounted on the postive lead between the batteries and the Arduino (this will likely involve cutting the wire and inserting the switch). The LED is mounted using an LED mount and glued in place. The sensor is pushed through from the inside (so the connecting pins are inside the enclosure) and glued in place. For extra security and to maintain neatness inside the enclosure cables may be secured using screws and thin sheets of flexible plastic. The enclosure ready for the Arduino board is shown in Fig. 11.

**Figure 12.**
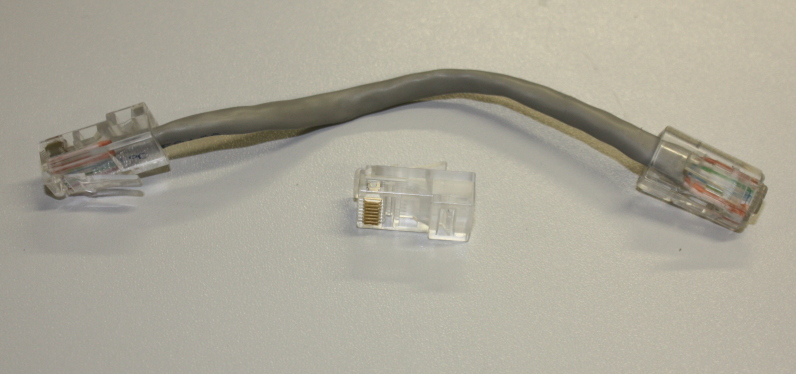
Custom made short Ethernet cable for connecting the Arduino Ethernet shield to the weatherproof Ethernet connector.

The connection between the Arduino's Ethernet shield and the weatherproof Ethernet connection requires a short length of Ethernet cable (Fig. 12). For neatness I created a short (c. 12 cm) cable using CAT V cable, two Ethernet sockets and the appropriate crimping tool. This cable should be in the 'patch' rather than crossover configuration (see, e.g.: http://www.learningelectronics.net/circuits/home-network-for-adsl.html). A short commercially available length of Ethernet cable will be less neat but equally functional.

**Figure 13.**
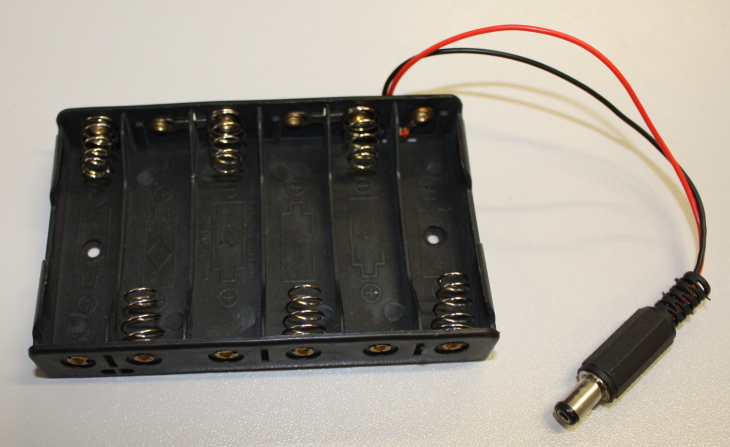
Battery holder for 6 AA batteries with Xmm connector for attaching to Arduino board.

Until this point it is likely you have been powering the device using the USB interface. Now that it is ready for use as a stand-alone data logger an alternative power source is needed. The Arduino has an in-built power regulator circuit that protects the delicate electronics from damage, and allows it to operate on a range of power supply voltages. It is possible to use 4 or 6 AA batteries to power the device. In order to use the built-in regulator this power must be supplied through the circular power adaptor on the board. This connector has a diameter of 2.1 mm and is centre pin positive. Battery holders with this connector can be purchased (Fig. 13) or built from a standard battery pack and power connector (available from electronic component retailers).

**Figure 14.**
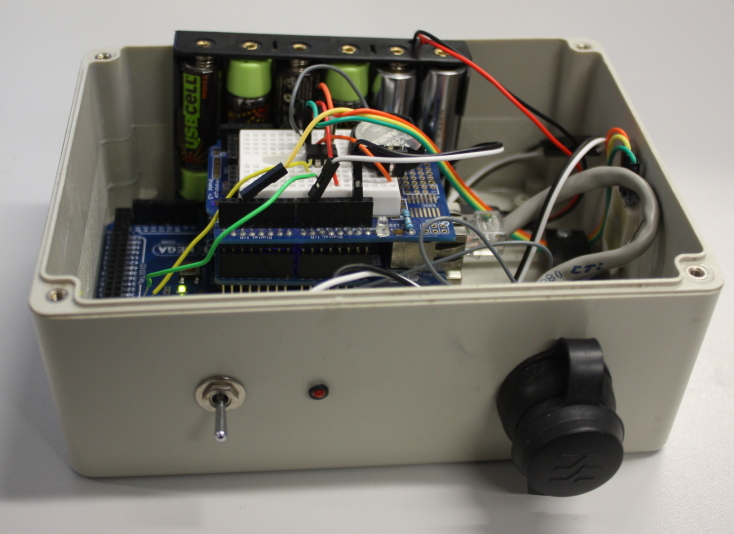
A completed device packaged in a watertight enclosure (with lid removed).

**Figure 15.**
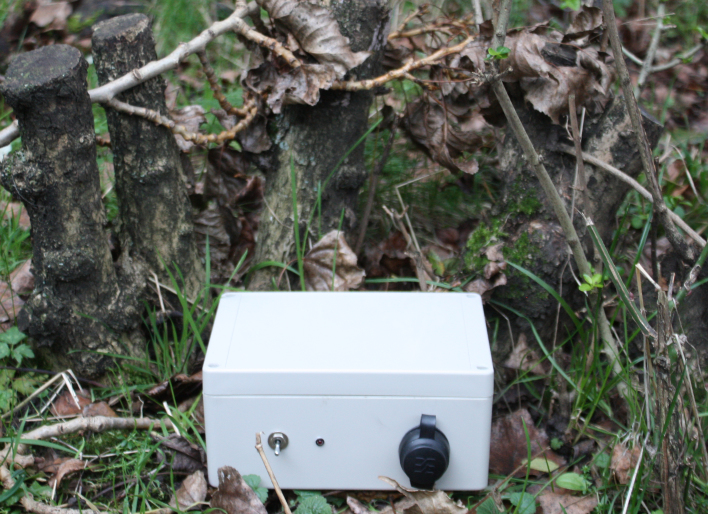
A complete unit monitoring the environment in the Wildlife Garden of the Natural History Museum, London.

A completed device, in an enclosure, is shown in Fig. 14 and Fig. 15.

### Use of the Scratchpads/Drupal module (basic)

The Arduino module for Drupal can be installed in the standard way for that platform, there are no other module dependencies. The settings for this software once installed can be found in the Configuration menu.

**Figure 16.**
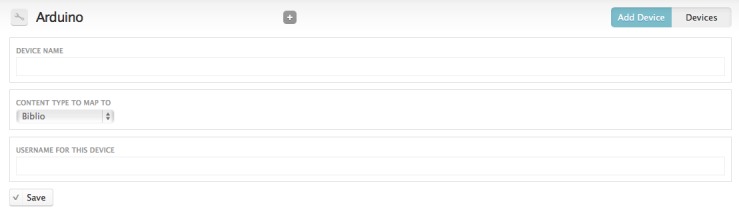
The 'Add Device' page for the Drupal module. The user must enter a human-readable name for the device (e.g. "Environment Monitor 1"), the Drupal content type that the data should be stored in, and the user name of a registered user on that site. Posts from the device will be recorded as being made from this user. As the new device is saved a machine-readable token is generated that uniquely identifies the device.

For a device to submit data to the website it must first be registered: from Configuration > Arduino select 'Add Device' (Fig. 16). This forms allows you to name the device, choose what Drupal content type the device will post data to (most likely the user will need to create a new custom content type for the data) and the Drupal user name of a user on the site. Once the device is saved a unique token is generated that uniquely identifies this device. This token is used by the device to identify itself to the website when it posts data.

**Figure 17.**
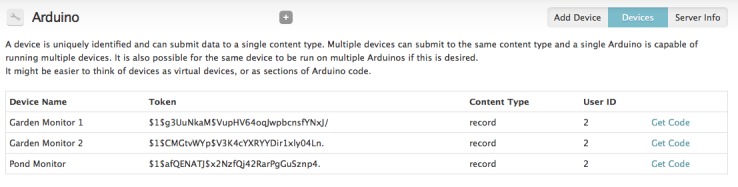
The 'Devices' page lists the devices registered with the website, the token they must use to submit data to this website, the content type that the data they submit should be stored in, the unique user identification of the user the devices will post content as and a link to get automatically generated Arduino code.

The 'Devices' page (Configuration > Arduino; Fig. 17) lists all of the devices that have been registered with the system, and also lists the generated tokens that the devices must use to submit data to this website.

Clicking on the 'Server Info' button will give a page with details the device will need to connect to the website it is being run on. These values, along with a token generated from the same website can be copy and pasted into the Arduino application downloaded from the repository for a quick setup.

Once the code (with modified values based on your server install) is uploaded to the Arduino the device will begin posting data to the server when it is turned on.

**Figure 18.**
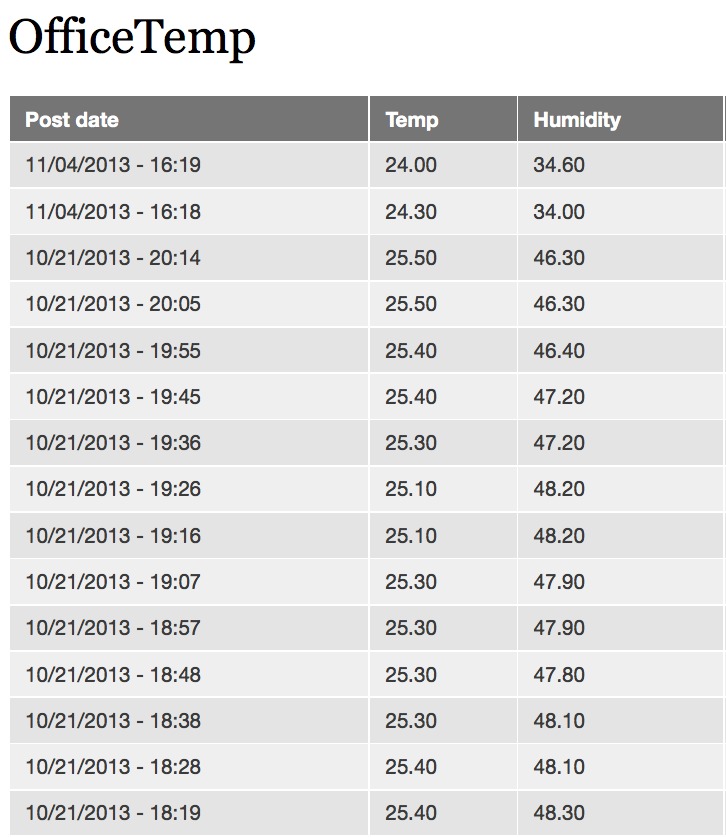
Table display of information posted from an Arduino to a Drupal website using the code and hardware described in this paper. The table display uses the Drupal Views module.

The information posted to the website from the device is now standard Drupal content and can be processed using standard Drupal modules. The most useful is likely to be the Views module which provides many options for querying and displaying content (Fig. 18).

### Use of the Scratchpads/Drupal module (advanced)

This section describes advanced use of the Drupal Arduino module that is not needed if you just wish to build the data logger and use it as described here. However if you wish to use the Drupal module as a tool for recording data from many sensors the 'virtual systems' section may be of use. If you wish to create your own custom Arduino project then the section 'Austomatic code generation' describes how the Arduino Drupal module can help you by generating the Arduino code needed to post to a Drupal content type from your device.

**Virtual systems** While most users of this software will just require the above configuration of one device posting data to one website the system is designed to allow greater flexibility than this. It is easy to give each device two or more different tokens so that they may post to several websites, or post as separate devices to one website. It is also possible to give multiple devices the same token and have them report data to one website. All devices sharing a token can be considered a virtual system with it's output as the website that generated that token. These virtual systems allow the same hardware to participate in multiple virtual systems. In most cases the device will not be continuously monitoring all variables, for example temperature measurements every couple of minutes is usually satisfactory. Instead of just performing empty cycles the device could be programmed to also perform data collection for another project using a different token (e.g. measuring the temperature, total dissolved solids, and light intensity at the bottom of a nearby pool of water).

**Figure 19.**
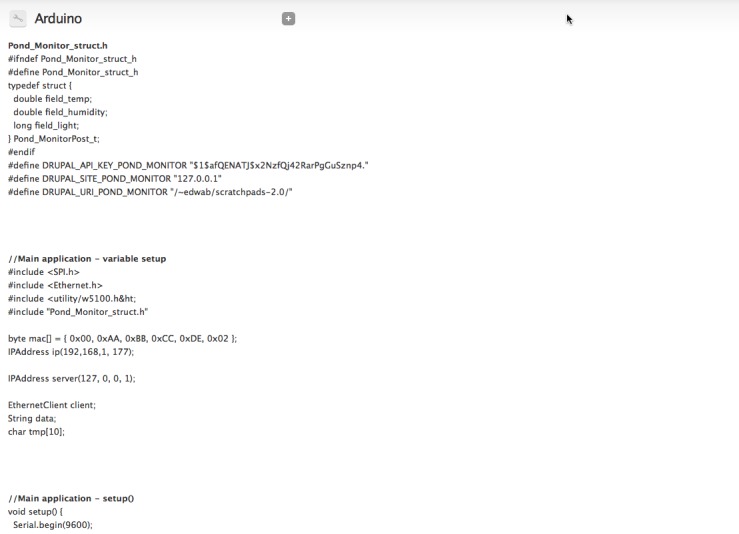
The code generated by the Drupal module's 'Get Code' functionality showing the full struct definition file (in this case Pond_Monitor_struct.h) and the start of the example code for the main application.

**Automatic code generation** If you wish to add the ability to post to a Drupal website to your own Arduino project this function of the Arduino module is designed to save you time and effort. The function is available for every device that you create on the Drupal site and automatically generates Arduino code for a suitable data structure and the code required to post data from the Arduino to Drupal. The automatic code generation function (Fig. 19) is available form the main 'Devices' page for every device created on the site. The code generator runs through the following processes to generate code that can be copy and pasted into the Arduino IDE:

- Find the definition of the Drupal content type associated with the device
- Generate a list of fields that can be posted to from the device
- For numeric fields check for min/max values and identify the smallest Arduino variable type that can be used (to save memory)
- Define a C struct in Arduino for holding the required information
- Generate an example program that accepts a populated C struct of the type defined above and posts it to the website that generated the code

To use the example code two files should be created in the Arduino IDE. One contains the struct definition and must use the file name ending in. h supplied in the example code. The other is the main application and can have any file name. Example application (Suppl. material 1) and header (Suppl. material 2) files are provided.

## Supplementary Material

Supplementary material 1 Example Arduino application generated by the 'Get Code' functionality Data type: Arduino source code
File: oo_5573.ino Ed Baker

Supplementary material 2 Example Arduino struct header file generated by the 'Get Code' functionality Data type: Arduino source code
File: oo_5574.h Ed Baker

## Figures and Tables

**Figure 9a.**
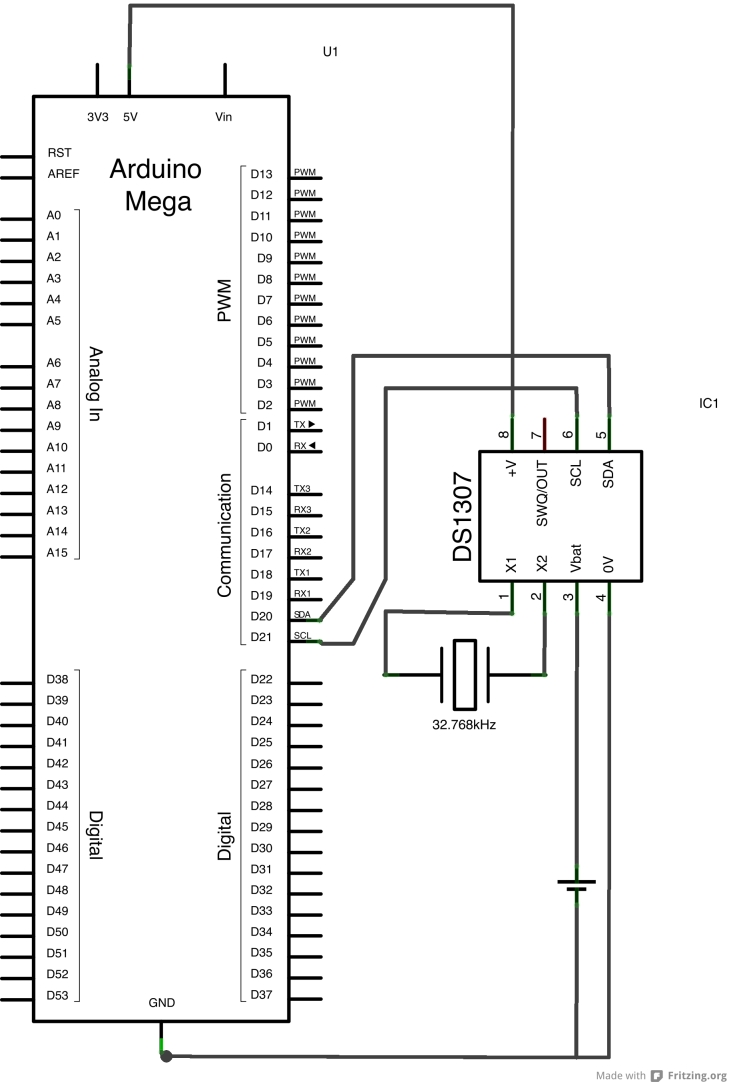
Schematic created using Fritzing.

**Figure 9b.**
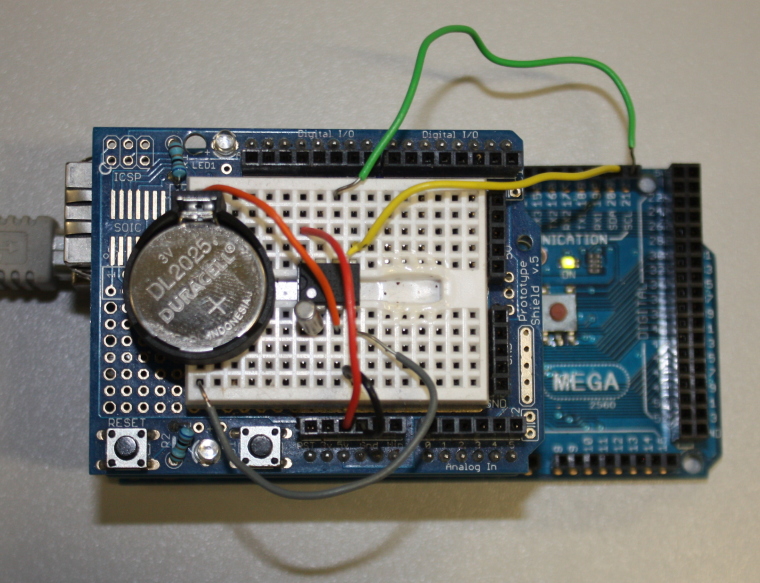
A real circuit created on the prototyping shield.
